# SPECT-CT Assessment of Pseudarthrosis after Spinal Fusion: Diagnostic Pitfall due to a Broken Screw

**DOI:** 10.1155/2013/502517

**Published:** 2013-09-18

**Authors:** Olivier Rager, Gaël Amzalag, Arthur Varoquaux, Karl Schaller, Osman Ratib, Enrico Tessitore

**Affiliations:** ^1^Nuclear Medicine Department, Geneva University Medical Center, Faculty of Medicine, University of Geneva, Rue Gabrielle-Perret-Gentil 4, 1211 Geneva, Switzerland; ^2^Department of Radiology, Geneva University Medical Center, Faculty of Medicine, University of Geneva, Rue Gabrielle-Perret-Gentil 4, 1211 Geneva, Switzerland; ^3^Department of Neurosurgery, Geneva University Medical Center, Faculty of Medicine, University of Geneva, Rue Gabrielle-Perret-Gentil 4, 1211 Geneva, Switzerland

## Abstract

A 43-year-old drug addicted female was referred for a L5-S1 posterolateral in situ fixation with autologous graft because of an L5/S1 severe discopathy with listhesis. After six months, low back pain recurred. A Tc-99m HDP SPECT-CT diagnosed a pseudarthrosis with intense uptake of the L5-S1 endplates and a fracture of the right S1 screw just outside the metal-bone interface without any uptake or bone resorption around the screw. The absence of uptake around a broken screw is a pitfall that the physician should be aware of.

## 1. Introduction

Spinal fusion is a common treatment for spinal instability [1]. Nevertheless, pseudarthrosis is a well-known long-term complication of spinal fusion. It is defined as a nonunion 6 months after surgery leading to a persistent movement in a specific spinal motion segment. Symptoms of pseudarthrosis may be nonspecific, and increasing local pain is by far the most common one. Diagnosis is often challenging, and some recent studies have shown that new hybrid imaging using SPECT-CT (Tc-99m HDP) [2, 3] or PET-CT (F-18 NaF) [4, 5] may improve diagnostic accuracy compared to CT scan alone.

## 2. Case Report

**Figure 1:**
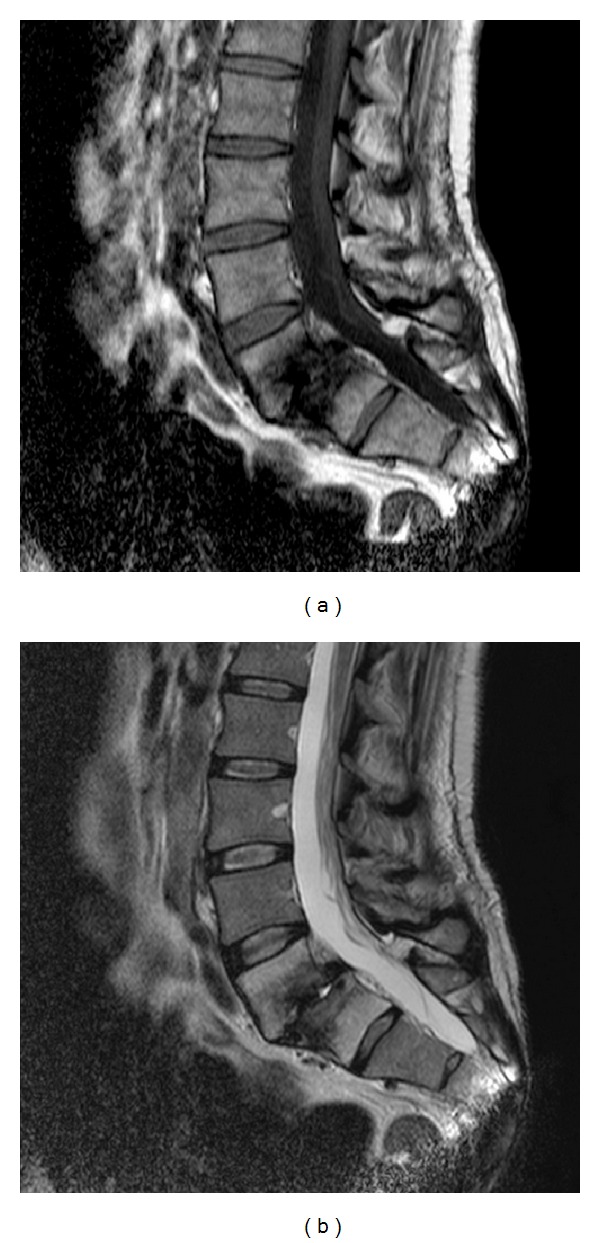
T1 (a) and T2 (b) sagittal lumbar MRI showing an L5/S1 severe discopathy with meyerding grade I listhesis and Modic type 3 sign.

**Figure 2:**
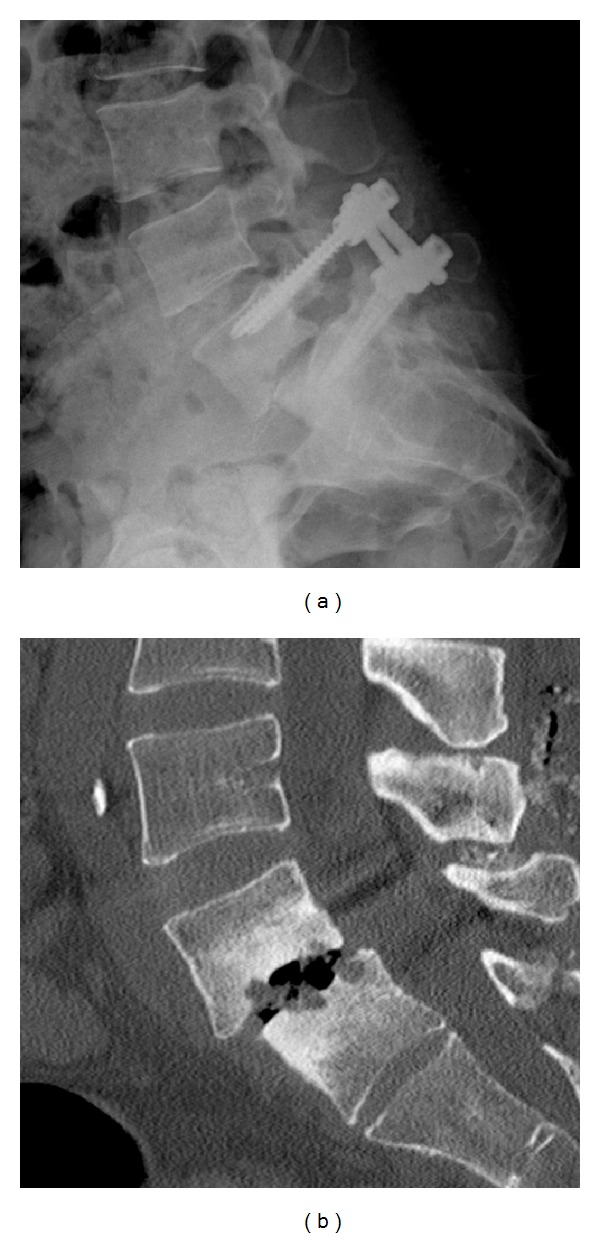
Lateral postop radiography (a) and postop CT scan (b) scan showed material in place, air in L5-S1 space, and reduction of the slippage L5-S1.

**Figure 3:**
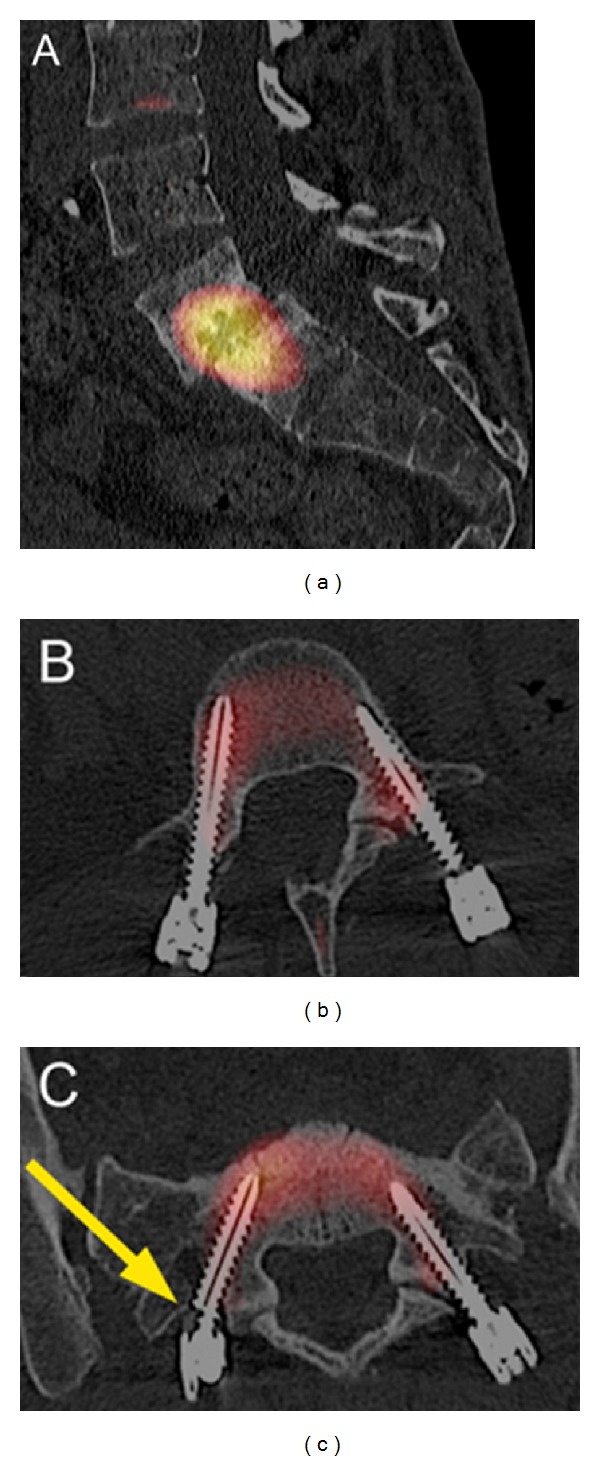
SPECT-CT revealed an intense uptake of the L5-S1 endplates (a) and a fracture of the right S1 screw without uptake and without bone resorption around the screw (c; arrow). The left S1 screw (c) and both L5 screws (b) showed no abnormalities, whereas the rods did.

A 43-year-old drug addicted female was referred to neurosurgeons for a chronic invalidating back pain and left side L5 sciatica, refractory to conservative treatment. A lumbar MRI showed an L5/S1 severe discopathy with Meyerding grade I listhesis and Modic type III sign (Figure 1). Surgery was limited to a L5-S1 posterolateral in situ fixation with autologous graft. Postop radiography and CT scan showed material in place and a reduction of the L5-S1 slippage (Figure 2). The patient was symptom-free for 6 months. After cracking without traumatism, low back pain recurred. A SPECT-CT was performed 7 months after surgery (Symbia T6, Siemens Healthcare, Germany) and 3 hours after injection of 800 MBq (21,6 mCi) of Tc-99m hydroxymethylene diphosphonate (HDP) (Figure 3). It revealed an intense uptake of the L5-S1 endplates characteristic of pseudarthrosis and a fracture of the right S1 screw without uptake and without bone resorption around the screw. The left S1 screw and both L5 screws showed no abnormalities, whereas the rods did. The neurosurgeons decided for a surgical revision with a transforaminal lumbar interbody fusion procedure, and the patient had an uneventful outcome.

## 3. Discussion

CT scan has supplanted plain radiography and has become the most used modality to detect pseudarthrosis even if it has been shown to have several limitations [6]. Despite have a very high sensibility, it falsely predicts pseudarthrosis in 8% of cases [7]. Some recent studies have shown that new hybrid imaging using SPECT-CT (Tc-99m HDP) or PET-CT (F-18 NaF) may improve diagnostic accuracy compared to CT scan alone. 

The assessment of pseudarthrosis requires the evaluation of both the posterior segment of the spine including screws and rods and the anterior segment with or without an interbody cage. Focal uptake in these sites is a characteristic finding of pseudarthrosis [2–5]. In our case, persistence of focal uptake in the anterior spinal segment permitted to identify pseudarthrosis. No uptake was observed around the fractured screw because the fracture occurred outside the metal-bone interface. By that time, the vertebral portion of the broken screw did not show any mobility or uptake. Nevertheless, this should not be considered a false negative of SPECT-CT. This is a pitfall that the physician should be aware of. Furthermore, the integrity of the material has to be carefully inspected on CT before analyzing the SPECT.

## 4. Conclusions

SPECT-CT is a useful tool for diagnosis of pseudarthrosis. It may show intense uptake at different levels as in the vertebral endplates and around screws or cages. The absence of uptake around a broken screw, as in our case, should not be considered as a false negative. CT alone should always be interpreted if clinical suspicion of pseudarthrosis exists.
